# Low circulating levels of neuregulin 4 as a potential biomarker associated with the severity and prognosis of obesity-related metabolic diseases: a systematic review

**DOI:** 10.1080/21623945.2024.2390833

**Published:** 2024-08-20

**Authors:** Khanyisani Ziqubu, Phiwayinkosi V. Dludla, Sinenhlanhla X.H. Mthembu, Bongani Nkambule, Sithandiwe E. Mazibuko-Mbeje

**Affiliations:** a Department of Biochemistry, North-West University, Mmabatho, South Africa; b Cochrane South Africa, South African Medical Research Council, Tygerberg, South Africa; c Department of Biochemistry and Microbiology, University of Zululand, KwaDlangezwa, South Africa; d School of Laboratory Medicine and Medical Sciences, University of KwaZulu-Natal, Durban, South Africa

**Keywords:** Neuregulin 4, brown adipose tissue, obesity, diabetes mellitus, cardiovascular diseases, non-alcoholic fatty liver disease

## Abstract

**Background:**

Neuregulin 4 (Nrg4) is a brown adipose tissue-derived adipokine that greatly affects systemic metabolism and improves metabolic derangements. Although abnormal circulating levels of Nrg4 are common in obesity, it remains elusive whether low or elevated levels of this batokine are associated with the onset of metabolic diseases.

**Aim:**

To assess Nrg4 levels and its role as a feasible biomarker to predict the severity of obesity, gestational diabetes mellitus (GDM), type 2 diabetes mellitus (T2DM), non-alcoholic fatty liver disease (NAFLD), and cardiovascular diseases (CVD).

**Methods:**

A search for relevant studies was performed systematically using prominent search engines, including PubMed, Google Scholar, and Embase, by following PRISMA guidelines.

**Results:**

Ample clinical evidence reported low serum/plasma levels of Nrg4 in obesity and these were inversely proportional to the indices of metabolic syndrome, including body mass index, waist circumference, triglycerides, fasting plasma glucose, and homoeostatic model assessment for insulin resistance as well as high-sensitivity C-reactive protein. Low circulating Nrg4 levels may aid in the prediction of morbid obesity, and subsequent GDM, T2DM, NAFLD, and CVD.

**Conclusion:**

Current clinical evidence emphasizes that the circulating levels of Nrg4 are decreased in morbid obesity, and it also highlights that Nrg4 May serve as a potential prognostic biomarker for obesity-related metabolic diseases.

## 1. Introduction

Metabolic diseases such as diabetes mellitus and cardiovascular disease (CVD) constitute the major parts of non-communicable diseases, which significantly contribute to premature deaths worldwide [1]. Poor health patterns, including an unhealthy diet and reduced physical inactivity, are likely contributors to the surge in metabolic diseases [2,3]. The consortium of metabolic abnormalities including insulin resistance, hyperglycaemia, dyslipidemia, and hypertension may give rise to metabolic syndrome (MetS) which often precedes the onset of type 2 diabetes (T2DM) and CVD [4,5]. Manifestation of MetS through ectopic fat accumulation can also severely affect the liver, resulting in the development of non-alcoholic fatty liver diseases (NAFLD) [6]. The negative effects of ectopic lipid accumulation may increase the risk of gestational diabetes mellitus (GDM) in women without diabetes during pregnancy [7,8]. Like the pathological consequences of T2DM [9], GDM is associated with deteriorated metabolic health and poor health patterns in pregnant women [10,11]. The clinical features of MetS include abnormal levels of low or high-density lipoprotein-cholesterol (LDL-c/HDL-c), including aberrant concentrations of triglycerides (TG), and fasting plasma glucose (FPG), as well as elevated high blood pressure (BP) [12]. Moreover, body mass index (BMI) and waist circumference (WC) are also deemed relevant to assess the state of MetS or obesity [13]. Individuals who meet at least three out of five diagnostic criteria for MetS are considered to have a high risk of developing T2DM and CVD [14].

In recent years, adipose tissue-derived bioactive molecules, known as adipocytokines or adipokines, have been increasingly studied as potential biomarkers for obesity and MetS [15–17]. For example, the ratio of leptin to adiponectin, which are two prominently studied adipokines [18], can be used to predict the occurrence of insulin resistance and even some instances the severity of MetS [19–21]. The use of both leptin and adiponectin has opened avenues for more research on the potential use of other adipokines to characterize obesity phenotype [22]. As such, brown adipose tissue (BAT) has emerged as a source of endocrine factors known as “batokines’ that exert powerful metabolic effects beyond regulating adaptive thermogenesis [23]. Amongst these endocrine factors, neuregulin 4 (Nrg4) from the epidermal growth factor family of extracellular ligands which signal via ErbB receptors of tyrosine kinases is gaining unwavering attention because of its potential modulatory role during the pathogenesis of obesity and other various metabolic diseases [24,25]. Briefly, Nrg4 is mainly expressed in BAT, whereas its presence is relatively low in other tissues like the skeletal muscle, liver, brain, and heart [26,27]. Experimental research shows that Nrg4 regulates diverse molecular mechanisms, including substrate metabolism, neurite outgrowth and angiogenesis [28], inflammation [29], hepatic lipogenesis [27], fuel oxidation [24], and beige fat thermogenesis [30]. Clinical research indicates that circulating levels of Nrg4 are significantly lower and negatively correlated with BMI and WC in patients with obesity and MetS [31]. Other elegant observational studies have also associated low Nrg4 levels with NAFLD [32] or coronary artery disease [33,34], and subclinical atherosclerosis in adults with obesity and MetS [33].

Extensively reviewed evidence has associated Nrg4 with protective effects against metabolic dysregulation in various metabolic diseases, such as insulin resistance, obesity, NAFLD, and diabetes mellitus through several mechanisms, such as anti-inflammation, autophagy regulation, pro-angiogenesis, and substrate metabolism [35,36]. However, cumulative studies have reported that Nrg4 levels are decreased during obesity, with an emerging body of evidence demonstrating its potential use as a biomarker for metabolic diseases. Thus, this systematic review aimed to elucidate the potential role of circulating levels of Nrg4 as a biomarker for the severity of obesity, GDM, T2DM, NAFLD, and CVD. This is essential for diagnostic or therapeutic approaches aimed at combating the devastating outcomes of metabolic diseases.

## 2. Methodology

The present systematic review was performed by following the Preferred Reporting Items for Systematic Reviews and Meta-Analysis (PRISMA) guidelines [37]. However, due to the diversity (heterogeneity) of included studies, only a qualitative analysis was done, without a meta-analysis. Although the current study does not have an approved protocol, online databases such as the Prospective Register of Systematic Reviews (PROSPERO) were accessed and screened to identify if any similar reviews were available and avoid duplication. Supplementary material 1 is attached, for the PRISMA checklist. Moreover, grey literature or ongoing relevant studies were searched and screened on http://www.opengrey.eu/ and http://www.ntis.gov/ to identify any relevant studies

### 2.1. Search strategy

A comprehensive search of eligible studies was conducted by two independent investigators on PubMed, Google Scholar, and Embase databases from inception to February 2024. The search was performed using the following search strategy: (‘neuregulin 4’ OR ‘Nrg4’ OR ‘Nrg-4’) AND (‘obesity’ OR (‘diabetes’ OR ‘diabetes mellitus’) OR (‘gestational diabetes’ OR ‘gestational diabetes mellitus’ OR ‘GDM’) OR (‘type 2 diabetes’ OR ‘type 2 diabetes mellitus’ OR ‘T2DM’ OR ‘diabetes mellitus’) OR (‘non-alcoholic fatty liver disease’ OR ‘NAFLD’ OR ‘fatty liver disease’) OR (‘cardiovascular diseases’ OR ‘CVD’) OR (‘metabolic disease’ OR ‘metabolic syndrome’)). Language and study type or study outcomes were not restricted to ensure complete and comprehensive search results.

### 2.2. Eligibility criteria for study inclusion

All identified articles were screened for eligibility through their titles and abstracts, and subsequently, the reference lists were hand-examined for additional relevant papers. This was carried out by two independent reviewers, while any disagreements were resolved by argumentum. Studies were considered eligible when they met the following inclusion criteria: (i) study population comprising of patients or participants diagnosed with metabolic diseases, including obesity, GDM, T2DM, NAFLD, and CVD, which reported on circulating Nrg4 levels; and (ii) studies reporting the relationship between Nrg4 levels and diverse metabolic diseases. The study design, sample size, and geographical location were not restricted. Primary studies done on animals were excluded, mainly because the focus of the current review was on the potential role of this adipokine in human subjects. Moreover, we also excluded meta-analysis and literature reviews, however, these were screened for primary studies.

### 2.3. Data extraction and quality evaluation

Two reviewers independently extracted the following data items from each eligible study: author name, year of publication, study site/region of publication, sample size, sex/gender, age range, and the main findings involving the regulation of Nrg4 in participants with MetS. Subsequently, the quality of all included studies was evaluated using the Newcastle-Ottawa Scale (NOS) [38], which is a comprehensive tool that has been validated to assess the quality of non-randomized studies. In terms of rating, the NOS scoring system was used to determine the quality of the included studies (range, 0–9 stars). Studies with scores greater than six (>6) were regarded as high quality, whereas those below four (<4) were regarded as poor quality.

## 3. Results

### 3.1. An overview of included studies

**Figure 1.**
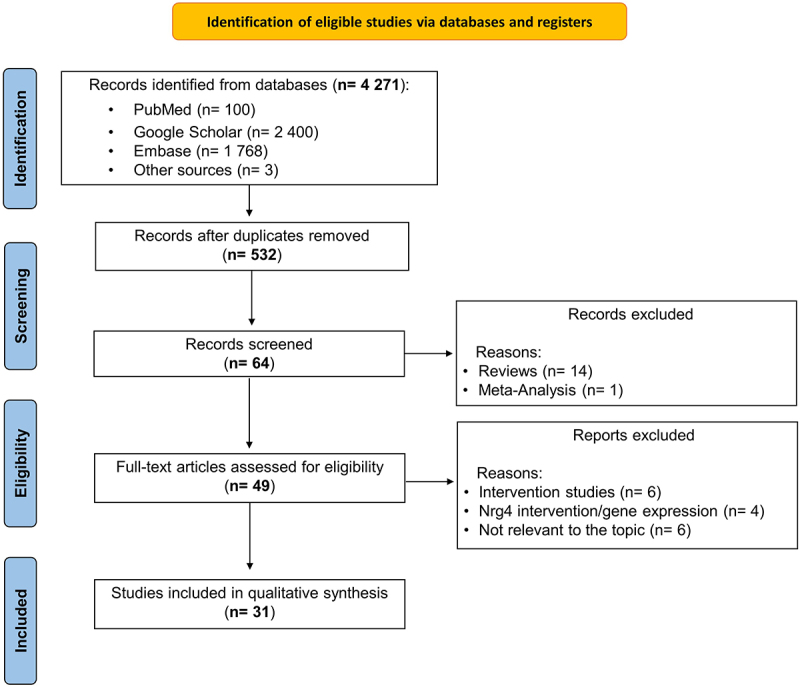
A representative flow diagram showing preferred reporting items for systematic reviews and meta-analysis (PRISMA) for study inclusion, with descriptive analysis for searching, screening, and final selection.

After searching and removing duplicates, a total of 4271 records were recovered from various search engines, as shown in Figure 1. Amongst these, five-hundred and thirty-two (*n* = 532) records were retained after removing duplicates. The final screening yielded forty-nine (*n* = 49) full-text articles that were potentially eligible. Finally, a total of thirty-one (*n* = 31) observational studies were found eligible after assessing the titles and abstracts based on the predetermined inclusion and exclusion criteria.

### 3.2. Characteristic features of included studies and their quality assessment

Our systematic search yielded thirty-one (*n* = 31) studies reporting the circulating levels of Nrg4 in participants with obesity and MetS (*n* = 4), NAFLD (*n* = 4), GDM (*n* = 7), T2DM (*n* = 12), and CVD-related disorders (*n* = 4) (Tables 1–5). As outlined in the Tables, observational studies, including cohort, case-control, and cross-sectional studies quantified Nrg4 levels either in plasma or serum using enzyme immunoassay (EIA) and mostly enzyme-linked immunosorbent assay (ELISA), which are analytical or diagnostic tools that are widely used in medicine and biomedical research for the detection and quantification of specific antigens or antibodies in a given sample [39]. Regarding geographic distribution, twenty-three studies were conducted in Asia (China, *n* = 16; Iran, *n* = 3; Iraq, *n* = 2; South Korea, *n* = 1; Pakistan, *n* = 1), four studies were conducted in Europe (Germany, *n* = 2; Spain, *n* = 1; Netherlands, *n* = 1), and four studies were from Turkey (*n* = 4).

**Table 1.** Clinical studies reporting on circulating neuregulin 4 (Nrg4) levels and their potential role in obesity.

Author, year	Country	Study populations	Study design	Sample	Assay	Outcomes
Cai et al. 2016 [31]	China	781 participants with obesity and MetS, at an average age of 54 years (32% male)	Cross-sectional	Serum	ELISA	Low levels of Nrg4 were accompanied by raised fasting plasma glucose and blood pressure. It was negatively correlated with BMI and WC but there was no association with triglycerides and HDL-c
Su-su et al. 2019 [41]	China	192 participants with metabolically unhealthy obesity, at an average age of 11 years (72% male)	Cross-sectional	Serum	ELISA	Low levels of Nrg4 were negatively correlated with BMI, waist-height ratio, and markers of liver injury ALT, and AST
Guo et al. 2021 [40]	China	781 participants with obesity and MetS, at an average age of 54 years (32% male)	Cross-sectional	Serum	ELISA	Low levels of Nrg4 were associated with WC, visceral fat level, and MetS
Martínez et al. 2022 [42]	Spain	55 participants with obesity, at an average age of 44 years (29% male)	Case-control	Serum	ELISA	High levels of Nrg4 were negatively correlated with insulin sensitivity and positively correlated with inflammatory maker hs-CRP

Abbreviations: ALT, alanine transaminase; AST aspartate transaminase; BMI, body mass index; HDL-c, high-density lipoprotein-cholesterol; hs-CRP, high-sensitivity C-reactive protein; ELISA, enzyme-linked immunosorbent assay; MetS, metabolic syndrome; Nrg4, neuregulin 4; WC, waist circumference.

**Table 2.** Clinical studies reporting on circulating neuregulin 4 (Nrg4) levels and their potential role in non-alcoholic fatty liver disease.

Author, year	Country	Study populations	Study design	Sample	Assay	Outcomes
Dai et al. 2015 [32]	China	87 participants with NAFLD, at an average age of 52 years (61% male)	Case-control	Serum	ELISA	Low levels of Nrg4 were associated with increased NAFLD
Wang et al. 2019 [50]	China	58 participants with NAFLD, at an average age of 11 years (71% male)	Cross-sectional	Serum	ELISA	Low levels of Nrg4 were inversely correlated with BMI, WC, triglycerides, fasting plasma insulin, and HOMA-IR
Tutunchi et al. 2021 [51]	Iran	50 participants with newly diagnosed NAFLD, at an average age of 50 years (56% male)	Case-control	Serum	ELISA	Low levels of Nrg4 were inversely correlated with body mass index, waist circumference, and triglycerides as well as HOMA-IR
De Munck et al. 2021 [52]	Netherlands	65 participants with NAFLD, at an average age of 40 years (48% male)	Cross-sectional	Plasma	ELISA	Nrg4 levels were not significantly affected

Abbreviations: BMI, body mass index; ELISA, enzyme-linked immunosorbent assay; HOMA-IR, homoeostatic model assessment for insulin resistance; NAFLD, non-alcoholic fatty liver disease; Nrg4, neuregulin 4; NR, not reported; WC, waist circumference.

**Table 3.** Clinical studies reporting on circulating neuregulin 4 (Nrg4) levels and their potential role in gestational diabetes mellitus.

Author, year	Country	Study populations	Study design	Sample	Assay	Outcomes
Kurek Eken et al. 2017 [54]	Turkey	63 participants with GDM, at an average age of 30 years (0% male)	Prospective cross-sectional	Serum	ELISA	High levels of Nrg4 were positively correlated with BMI, FPG, HOMA-IR, triglycerides, and low-density lipoprotein-cholesterol, but negatively correlated with HDL-c
Kralisch et al. 2018 [55]	Germany	74 participants with GDM, at an average age of 31 years (0% male)	Prospective cohort	Serum	ELISA	Low levels of Nrg4 were positively and independently associated with irisin, but not other BAT-secreted adipokines
Zhang et al. 2021 [56]	Germany	36 participants with GDM, at an average age of 30 years (0% male)	Cross-sectional	Serum	ELISA	Low levels of Nrg4 were negatively correlated with FPG, HOMA-IR, interleukin 6, leptin, tumor necrosis factor-alpha, and monocyte chemoattractant protein-1, and positively correlated with HDL-c
Attique et al. 2022 [57]	Pakistan	37 participants with GDM, at an average age of 30 years (0% male)	Cross-sectional	Serum	ELISA	Low levels of Nrg4 had a direct weak association with HOMA-IR, inverse relationship with cholesterol and low-density lipoprotein but a significant association with insulin
Li et al. 2022 [59]	China	58 participants with GDM, at an average age of 30 years (0% male)	Cross-sectional	Serum	ELISA	Low levels of Nrg4 were closely related to insulin resistance
Cindoglu et al. 2023 [58]	Turkey	34 participants with GDM, at an average age of 30 years (0% male)	Case-control	Serum	ELISA	Low levels of Nrg4 did not correlate with thiol-disulfide balance, an indicator of oxidative stress
Al-Bayati and Saleh 2023 [61]	Iraq	45 participants with GDM, at an average age of 35 years (0% male)	Case-control	Serum	ELISA	Low levels of Nrg4 were negatively associated with FPG and HOMA-IR, while positively associated with high-density lipoprotein

Abbreviations: BMI, body mass index; ELISA, enzyme-linked immunosorbent assay; FPG, fasting plasma glucose; GDM, gestational diabetes mellitus; HDL-c, high-density lipoprotein cholesterol; HOMA-IR, homoeostatic model assessment for insulin resistance; LDL-c, low-density lipoprotein-cholesterol; Nrg4, neuregulin 4.

**Table 4.** Clinical studies reporting on circulating neuregulin 4 (Nrg4) levels and their potential role in type 2 diabetes mellitus.

Author, year	Country	Study populations	Study design	Sample	Assay	Outcomes
Kang et al. 2016 [63]	South Korea	57 participants with T2DM, at an average age of 51 years (53% male)	Case-control	Serum	ELISA	High levels of Nrg4 were correlated with FPG and insulin resistance, but not with HbA1c
Chen et al. 2017 [64]	China	96 participants with diabetes, at an average age of 59 years (36% male)	Case-control	Serum	ELISA	High levels of Nrg4 were correlated with FPG, BMI, WC, HC, NC, ALT, HDL-c, TC, uric acid, and eGFR
Zhang et al. 2017 [66]	China	103 participants with T2DM, at an average age of 52 years (62% male)	Cross-sectional	Serum	ELISA	Low levels of Nrg4 were negatively correlated with FPG, fasting insulin, and HOMA-IR, while positively correlated with HDL-c, but not associated with WC, HC, BMI
Yan et al. 2017 [67]	China	310 participants with T2DM, at an average age of 54 years (48% male)	Cross-sectional	Plasma	ELISA	Low levels of Nrg4 were inversely associated with WBC, and hs-CRP but positively associated with HDL-c
Yan et al. 2018 [68]	China	178 participants with T2DM with metabolic syndrome, at an average age of 54 years (47% male)	Cross-sectional	Plasma	ELISA	Low levels of Nrg4 were positively correlated with HDL-c and apolipoprotein A but negatively correlated with TC, hs-CRP, gamma-glutamyl transferase levels, and neutrophil and WBC count
Kocak et al. 2019 [65]	Turkey	100 participants with T2DM, at an average age of 59 years (49% male)	Cross-sectional	Serum	ELISA	High levels of Nrg4 were significantly correlated with FPG but not HbA1c
Yan et al. 2019 [70]	China	66 participants with T2DM and DPN, at an average age of 65 years (59% male)	Cross-sectional	Plasma	ELISA	Low levels of Nrg4 were negatively correlated with anthropometric, biochemical, and clinical parameters, including 8-iso-prostaglandin F2α, VPT, and hs-CRP
Yan et al. 2020 [71]	China	76 participants with T2DM and DPN, at an average age of 64 years (61% male)	Cross-sectional	Plasma	ELISA	Low levels of Nrg4 were negatively associated with VPT
Kocak et al. 2020 [72]	Turkey	50 participants with T2DM and diabetic microvascular complications, at an average age of 61 years (54% male)	Cross-sectional	Serum	ELISA	Low levels of Nrg4 were negatively correlated with FPG, HbA1c, and microalbuminuria
Kamal et al. 2022 [69]	Iraq	30 participants with T2DM and DPN, at an average age of 61 years (54% male)	Cross-sectional	Serum	ELISA	Low levels of Nrg4 were associated with DPN
Zhong et al. 2023 [73]	China	77 participants with T2DM and coronary artery disease, at an average age of 60 years (55% male)	Case-control	Serum	ELISA	Low levels of Nrg4 were negatively correlated with a history of hypertension, BMI, FPG, HbA1c, TG, and triglyceride glucose index, but positively correlated with HDL-c
Ding et al. 2023 [74]	China	55 participants with T2DM and early diabetic nephropathy, at an average age of 53 years (35% male)	Prospective cohort	Plasma	ELISA	Low levels of Nrg4 were positively correlated with TC, serum creatinine, urinary albumin/creatinine ratio, low-density lipoprotein, triglyceride, blood urea nitrogen, and blood uric acid, but negatively correlated with eGFR and HDL

Abbreviations: ALT, alanine transaminase; BMI, body mass index; DPN, diabetic peripheral neuropathy; ELISA, enzyme-linked immunosorbent assay; eGFR, estimated glomerular filtration rate; FPG, fasting plasma glucose; HbA1c, glycated haemoglobin A1c; HC, hip circumference; HDL-c, high-density lipoprotein-cholesterol; HOMA-IR, homoeostatic model assessment for insulin resistance; hs-CRP, high sensitivity C-reactive protein; NC, neck circumference; Nrg4, neuregulin 4; TC, total cholesterol; T2DM, type 2 diabetes mellitus; VPT, vibration perception threshold; WBC, white blood cell; WC, waist circumference. ^a^Values are all given as a group with metabolic disease/control group.

**Table 5.** Clinical studies reporting on neuregulin 4 (Nrg4) levels and their potential role in participants with cardiovascular diseases.

Author, year	Country	Study populations	Study design	Sample	Assay	Outcomes
Jiang et al. 2016 [33]	China	485 participants with obesity at high CVD risk, at an average age of 54 years (27% male)	Cross-sectional	Serum	ELISA	Low levels of Nrg4 were negatively associated with subclinical atherosclerosis or CVD
Tian et al. 2019 [34]	China	47 participants with CAD and high SYNTAX score at an average age of 67 years (18% male)	Cross-sectional	Plasma	EIA	Low levels of Nrg4 were inversely associated with the presence and severity of CAD
Rahimzadeh et al. 2020 [78]	Iran	144 participants with acute coronary syndrome, at an average age of 46 years (55% male)	Case-control	Serum	ELISA	Low levels of Nrg4 were negatively correlated with HDL-c
Alipoor et al. 2023 [79]	Iran	76 participants with CAD, at an average age of 61 years (74% male)	Cross-sectional	Serum	ELISA	Nrg4 levels were unaltered, and they were not associated with the odds of having CAD

Abbreviations: ELISA, enzyme-linked immunosorbent assay; CVD, cardiovascular disease; CAD, coronary artery disease; HDL-c, high-density lipoprotein cholesterol; Nrg4, neuregulin 4.

### 3.3. Quality of included studies

In terms of the quality assessment based on the NOS rating system, the included studies scored moderate to high quality of evidence (Supplementary material 2). Almost all these studies were observational, and cross-sectional in nature. Notably, the quality of observational studies depends on meticulous design, rigorous data collection, and robust statistical methods, allowing them to complement experimental research and contribute valuable insights to the scientific community. The below sections describe the modulation of Nrg4 during the pathogenesis of diverse metabolic complications.

### 3.4. Low circulating levels of Nrg4 were associated with an increased risk of metabolic syndrome in patients with obesity

Obesity and MetS are pressing global health issues, with biomarkers like adipokines potentially offering valuable insights into the pathogenesis of these conditions. Thus, measuring the levels of circulating adipokines like Nrg4 remains crucial to unveil their role in the development and progression of obesity to MetS [15–17].

Our systematic search yielded four (*n* = 4) studies specifically reporting on the modulation of Nrg4 in individuals with obesity and MetS (Table 1). These include one case-control and three cross-sectional studies assessing Nrg4 levels in the serum. A cross-sectional study by Cail et al. [31] found that adults with obesity and MetS had lower levels of circulating Nrg4 and a high prevalence of raised FPG and BP. These Nrg4 levels were negatively correlated with WC and BMI, but there was no association with raised triglycerides and reduced HDL-c [31]. Importantly, clinical characteristics by quartiles of serum Nrg4 levels in obese subjects revealed that the prevalence of MetS was significantly higher in participants with lower levels of serum Nrg4 compared to those with the highest values [31]. In the same population, Guo et al. [40] conducted a community-based cross-sectional study to examine and quantify the degree of the mediation effect of circulating Nrg4 on the association between obesity and MetS. It was found that the indices of adiposity and MetS are linked through circulating Nrg4 levels, suggesting that circulating Nrg4 might be a potential predictor of MetS. Similar to the adult population, a cross-sectional study by Su-su et al. [41] found that low circulating Nrg4 levels were negatively associated with fat mass, waist-height ratio (WHtR), and degree of metabolic disease in children with obesity.

As opposed to the outcomes of cross-sectional studies, a case-control study performed by Martínez et al. [42] reported that the serum levels of Nrg4 were elevated in participants with obesity and insulin resistance, and it was positively associated with insulin resistance and high-sensitive C-reactive protein (hs-CRP), but not with the markers of liver injury. However, cultured hepatocyte HepG2 treated with human recombinant Nrg4 displayed decreased gluconeogenic- and mitochondrial biogenesis-related gene expression and reduced mitochondrial respiration [42]. Taken together, both cross-sectional and case-control studies emphasized the health-promoting effect of Nrg4 while cross-sectional studies also demonstrated that low circulating levels of Nrg4 hold potential as a predictive factor for developing MetS. However, this requires well-designed prospective studies with longitudinal assessment of Nrg4 levels to resolve any discrepancies about the causality.

### 3.5. Low circulating levels of Nrg4 were associated with increased adiposity, insulin resistance, and non-alcoholic fatty liver disease

NAFLD is the most common chronic liver disease [43], which is closely associated with the components of obesity, T2DM, and MetS [44–46]. For example, excessive fat deposition in the liver occurs concurrently with elevated obesity indices as well as metabolic risk factors, such as BMI, WC, dyslipidaemia, hyperglycaemia, and insulin resistance [47]. Emerging evidence indicates that the liver and BAT are involved in the interorgan crosstalk via Nrg4, and this may play a role in the pathogenesis of liver diseases [48,49]. In rodents, Nrg4 inhibits *de novo* hepatic lipogenesis which in turn suppresses the progression of NAFLD to non-alcoholic steatohepatitis [24].

In this review, four (*n* = 4) studies have reported the association between circulating levels of Nrg4 and NAFLD (Table 2). An equal part of these studies includes case-control and cross-sectional studies quantifying Nrg4 in both serum and plasma. A case-control study by Dai et al., [32] reported that serum Nrg4 levels were significantly decreased in adults with NAFLD compared to those without this condition. In general, hepatic insulin resistance contributes to NAFLD directly by increasing *de novo* lipogenesis and indirectly by increasing free fatty acid flux. To support this notation, cross-sectional and case-control studies conducted by Wang et al. [50] and Tutunchi et al. [51] found that low serum Nrg4 levels were inversely correlated with obesity indices (BMI, WC, and WHtR) and glucose and lipid metabolic indices (TG, FPI, and HOMA-IR) in children and adults with NAFLD, respectively. To uncover the association of Nrg4 with the severity of NAFLD, clinical characteristics by quartiles of serum Nrg4 levels [32,51], and odds ratios for NAFLD [50] were assessed in adults and children, respectively. It was found that circulating Nrg4 levels play an utmost important role in the pathogenesis of NAFLD preceded by increased adiposity and insulin resistance, as well as identifying children with obesity at high risk for NAFLD.

Contrarily to serum Nrg4 levels, a cross-sectional study by de Muncket al., 2021 determined the levels of Nrg4 in the plasma and its association with fatty liver disease severity, where it was found that Nrg4 levels were not significantly different between NAFLD subjects with or without hepatic fibrosis [52]. In view of the above outcomes, the contribution of circulating Nrg4 in the development of NAFLD requires further verification in prospective cohort studies with larger sample sizes in order to adequately explore its role as a risk biomarker. Nonetheless, circulating Nrg4 levels hold great potential in predicting the pathobiology of NAFLD in children and adults with obesity.

### 3.6. Low circulating levels of Nrg4 were associated with increased adiposity, inflammation, and insulin resistance in women with gestational diabetes mellitus

Gestational diabetes mellitus (GDM) is the most common metabolic disturbance during pregnancy, and it is set to increase in parallel with the global obesity epidemic [53]. In fact, GDM may lead to maternal T2DM and potential adverse cardiometabolic complications in the offspring [53]. Although GDM is associated with an increased risk of T2DM, few studies have evaluated the correlation or predictive nature of adipokines during the pathogenesis of GDM during and after pregnancy.

In this systematic review, seven (*n* = 7) studies have demonstrated the association between the circulating Nrg4 levels and GDM (Table 3). These studies include one prospective cohort study, two case-control studies, and four cross-sectional studies assessing the levels of Nrg4 in the serum. While a prospective cross-sectional study by Kurek Eken et al. [54] reported that serum Nrg4 levels were significantly elevated in women with GDM compared to healthy subjects, a prospective cohort study by Kralisch et al. [55] reported the opposite outcomes showing that serum Nrg4 levels were low and improved during pregnancy and at the postpartum time point in women with GDM, respectively. In agreement with the latter, many cross-sectional and case-control studies have reported that serum Nrg4 levels were significantly low in women with GDM [55–59].

In general, oxidative stress and inflammation are key pathological processes in the development of diabetes [60]. A cross-sectional study by Zhang et al. 2021 [56] found that low serum Nrg4 levels were negatively associated with FPG, HOMA-IR and leptin as well as pro-inflammatory cytokines interleukin 6 (IL-6), tumour necrosis factor-alpha (TNF-α), and monocyte chemoattractant protein-1 (MCP-1) in GDM. In a recent case-control study conducted by Cindoglu et al. [58], there was no correlation between low serum Nrg4 levels and thiol/disulphide homoeostasis as an indicator of oxidative stress in women with GDM. In both cross-sectional and case-control studies, it was reported that low serum Nrg4 levels were negatively associated with insulin resistance ‘HOMA-IR’ in women with GDM, suggesting an increased risk of T2DM [59,61]. It is speculated that the increase in circulating Nrg4 levels postpartum can be attributed to reduced insulin resistance. Thus, the current clinical evidence revealed that low circulating levels of Ngr4 contribute to the development of diabetes, and it may play a role in predicting the occurrence of diabetes in pregnancy. However, there are very limited prospective cohort studies to confirm these findings.

### 3.7. Low Nrg4 levels were associated with inflammation, oxidative stress, and insulin resistance in patients with type 2 diabetes mellitus

T2DM is the most predominant form of diabetes mellitus, which is marked by the state of insulin resistance [62]. In this review, twelve (*n* = 12) studies reported the association between the circulating levels of Nrg4 and T2DM (Table 4). These include one prospective cohort study, three case-control studies, and eight cross-sectional studies assessing Nrg4 levels in serum and plasma. Among the very first studies assessing the serum Nrg4 levels in people with diabetes, two case-control studies by Kang et al. [63] and Chen et al. [64] reported that serum Nrg4 levels were elevated in newly diagnosed T2DM, however, they were correlated with FPG but not glycated haemoglobin A1c (HbA1c). Similar outcomes were observed in a cross-sectional study conducted by Kocak et al. [65] found that high Nrg4 levels were closely correlated with glucose parameters and adiposity proxies, such as FPG, BMI, WC, hip circumference (HC), neck circumference (NC), total cholesterol (TC), HDL-c, uric acid, and estimated glomerular filtration rate (eGFR), however, this was speculated to be a protective response from metabolic dysregulations.

In contrast to few studies reporting elevated serum Nrg4 levels in T2DM, many observational studies including cross-sectional, case-control, and prospective cohort studies demonstrated that circulating levels of Nrg4 were low in individuals with T2DM. For instance, several cross-sectional studies, including Zhang et al. [66] reported that serum Nrg4 levels were decreased and negatively correlated with FPG, fasting insulin, and HOMA-IR in participants with T2DM, while studies by Yan and co-workers [67,68] found that low plasma Nrg4 levels were inversely correlated with proinflammatory mediators hs-CRP and white blood cell count but positively correlated with HDL-c. In subjects with T2DM and diabetic peripheral neuropathy (DPN), serum Nrg4 levels were low [69], and they were negatively correlated with markers of inflammation and oxidative stress like hs-CRP, 8-iso-prostaglandin F2α (8-iso-PGF2α), and vibration perception threshold (VPT) in the plasma [70,71].

Circulating levels of Nrg4 were assessed in the serum and plasma of patients with T2DM-related macrovascular complications such as nephropathy and coronary artery disease [72–74]. A cross-sectional study by Kocak et al. [72] found that participants with T2DM and diabetic microvascular complications had low serum levels of Nrg4 and were negatively correlated with FPG, HbA1c, and microalbuminuria [72]. In agreement with this cross-sectional study, a case-control study performed by Zhong et al. [73] found that patients with T2DM and coronary artery disease had significantly low serum Nrg4 levels which negatively correlated with a history of hypertension, BMI, FPG, HbA1c, TG, and TG-glucose, and positively correlated with HDL-c. These outcomes were further confirmed in a prospective cohort study conducted by Ding et al. [74] reported that low plasma levels of Nrg4 were positively correlated with TC, TG, LDL, serum creatinine, urinary albumin/creatinine ratio, blood urea nitrogen, and blood uric acid, but negatively correlated with eGFR and HDL in patients with T2DM and diabetic nephropathy. Collectively, a growing body of clinical evidence demonstrates that T2DM is associated with low circulating levels of Nrg4, as a potential predictive factor. However, there is only one prospective cohort study in this context of association between Nrg4 and T2DM, therefore well-designed prospective studies with larger sample sizes and long follow-up periods are warranted.

### 3.8. Low circulating levels of Nrg4 were associated with increased carotid intima-media thickness, subclinical atherosclerosis, and severity of coronary artery disease

CVD remains the leading cause of death, especially in patients with diverse metabolic complications [75]. Preclinical evidence has been consistently advocating for the cardioprotective effect of Nrg4 [76,77]. In this review, four (*n* = 4) studies have reported that circulating levels of Nrg4 are decreased in subjects with cardiometabolic complications, and this precedes severe cases of CVD (Table 5). These studies include one case-control and three cross-sectional studies assessing Nrg4 levels in both plasma and serum. A cross-sectional by Jiang et al. [33] found that low serum Nrg4 levels were associated with elevated carotid intima-media thickness, an early marker of CVD in individuals with obesity. Another cross-sectional by Tian et al. [34] also confirmed that the plasma Nrg4 levels were significantly reduced and negatively correlated with the presence and severity of the disease in patients with CAD [34]. A case-control study by Rahimzadeh et al. [78] reported that serum Nrg4 levels were significantly lower in patients with acute coronary syndrome compared to healthy individuals, with these subjects showing high levels of HDL-c contents [78]. However, a cross-sectional performed by Alipoor et al. 2023 [79] reported that serum Nrg4 levels were not associated with the odds of having CAD. Although these studies indicate that low circulating levels of Ngr4 are consistent with the development of CVD and suggest that Nrg4 might be a candidate marker of CVD risk, there is a lack of large-scale and prospective population-based studies to further verify these outcomes.

## 4. Clinical evidence reporting on the prognostic value of neuregulin 4 as a potential biomarker for obesity-related metabolic diseases

Starting with obesity, as a major pathology responsible for the development of the MetS. To elucidate the predictive value of circulating Nrg4 in obesity, Su-su et al. [41] analysed the receiver-operating characteristics (ROC) curves of circulating Nrg4 in children with obesity. In this study, Nrg4 was compared with other adipokines including adiponectin, a clinically relevant biomarker for the detection of MetS [41]. According to ROC analysis, the diagnostic cut-off points of serum Nrg4 and adiponectin for evaluating metabolic abnormalities were 5.5 ng/mL and 5.56 μg/mL, respectively [41]. Although Nrg4 had a low cut-off point, both Nrg4 and adiponectin levels were associated with the degree of metabolic disorders, suggesting the potential to predict the occurrence or risk of metabolic disease in children and adolescents with obesity. Contrarily, Nrg4 displayed efficient predictive scores, sensitivity, and specificity for GDM, T2DM, and CVD-related complications in the adult population, as discussed below.

The identification of maternal factors that could predict the adverse outcomes of metabolically compromised pregnancies could serve as valuable tools for early detection of high-risk pregnancies, facilitating close follow-up, and prevention of pregnancy complications such as GDM. Although extensive research has been conducted, investigated biomarkers have not yet achieved clinical applicability. Several studies have demonstrated that Nrg4 could be a potential diagnostic tool for GDM [56,57,61]. For example, Nrg4 combined with inflammatory cytokines such as IL-6 and TNF-α had better diagnostic ability for GDM with an area under the curve (AUC) value of 0.759 [56]. In terms of diagnostic efficiency, Li et al. 2022 demonstrated that Nrg4 had a cut-off threshold was 96.25 ng/ml and an AUC value of 0.626 with 66.7% sensitivity and 62.07% specificity for the diagnosis of GDM [57]. However, Nrg4 showed efficient sensitivity (91.11%) and specificity (66.67%) with the cut-off point with a positive predictive value of 73.21 and a negative predictive value of 88.23 [61].

To date, cumulative studies continued to explore the prognostic or predictive role of Nrg4 in T2DM and diabetic-related complications. For example, Kamal et al. [69] observed that Nrg4 levels were significantly reduced in diabetic patients with DPN and speculated that Nrg4 can be used as a potential biomarker for early detection of DPN. To verify this, the analysis of an AUC curve was 0.943 and Nrg4 was able to identify patients with DPN at a level of 0.862 ng/mL, a sensitivity of 86.7%, and a specificity of 83.3%. Subsequently, the predictive efficiency of Nrg4 was compared with 25-hydroxy vitamin D, a secosteroid hormone that regulates other neurotrophic factors and adipokines gene expression [71]. The results revealed that the best cut-off value for circulating Nrg4 to predict DPN was 3.03 ng/ml (sensitivity: 55.7%, specificity: 71.1%, and AUC 0.660), whereas the best cut-off value for circulating 25-hydroxy vitamin D to predict DPN was 16.491 ng/ml (sensitivity: 52.3%, specificity: 76.3%, and AUC 0.635) [71]. Recently, Ding et al. [74] hypothesized that the occurrence and progression of diabetic nephropathy can be early predicted by Nrg4 and homocysteine, an amino acid that enhances the production of several pro-inflammatory cytokines [74]. The AUCs predicted for diabetic nephropathy were Nrg4 (0.91, 95%CI: 0.859, 0.961), HCY (0.885, 95%CI: 0.822, 0.948), and homocysteine/NRG4 (0.961, 95%CI: 0.928, 0.994), respectively [74]. From these outcomes, it was concluded that combined detection of homocysteine/Nrg4 can be useful for early detection of diabetic nephropathy or diabetic kidney disease [74]. To explore the predictive value of circulating Nrg-4 for T2DM-CAD, the ROC curves of Nrg4 together with a glucogenic adipokine asprosin were analysed [73]. The results revealed that the best cut-off value for circulating asprosin to predict CAD was 19.0 ng/ml (sensitivity: 66.2%, specificity: 71.2%, and AUC 0.671), and the best cut-off value for circulating Nrg-4 to predict CAD was 11.175 ng/ml (sensitivity: 67.5%, specificity: 75%, and AUC 0.772) [73]. The results also revealed that there was a significantly improved diagnostic efficacy with the combination of Nrg4 and asprosin as marked by the sensitivity, specificity, and AUC of 64.9%, 81.2%, and 0.796, respectively [73].

Other elegant studies also uncovered the prognostic value of Nrg4 on the complications of CVD. Serum Nrg4 levels displayed a significantly high AUC value of 0.68 and 0.74 for detecting increased carotid intima-media thickness (CIMT) and atherosclerotic plaque in patients with obesity and high risk of subclinical cardiovascular disease [33]. Moreover, the cut-off points for Nrg4 levels were 0.71 ng/ml for detecting increased CIMT and 0.69 ng/ml for detecting the presence of carotid plaque [33]. Likewise, circulating Nrg4 levels were significantly reduced and negatively correlated with the presence and severity of the disease in patients with coronary artery disease [34]. The AUC values were 0.629 and 0.802 for assessing the presence and severity of coronary artery disease, respectively. Although the Nrg4 had 43.8% sensitivity and 96.9% specificity for identifying coronary artery disease, cross-sectional data showed that Nrg4 performed better in terms of sensitivity (73.1%) and specificity (87.3%) for identifying severe coronary artery lesions [34]. This was consistent in the case-control study where Nrg4 showed a significantly high area under the curve value (AUC, 0.85; 95% CI, 0.75 to 0.94) with 81.4% sensitivity and 95.3% specificity to identify coronary artery disease [80]. Taken together, Nrg4 performed better for predicting various metabolic disorders when compared to other well-recognized molecules that serve as feasible biomarkers, such as adiponectin, homocysteine, and 25-hydroxy vitamin D. These outcomes emphasize that Nrg4 could be a potential biomarker for the occurrence of obesity-related metabolic diseases, but also highlighted the association between low Nrg4 levels and worse visual outcomes and risk of concurrent MetS. However, the predictive role of Nrg4 on metabolic disease, particularly CVD should be compared with other conventional biomarkers such as C-reactive protein (CRP) for further investigations. Moreover, current observational studies assessing the correlation between Nrg4 levels, and the risk of developing obesity and metabolic disease are focused on the adult population, with limited data on children and adolescents. To properly utilize Nrg4 as a tool for identifying the risk of metabolic disease occurrence, people of all ages should be considered for future studies.

## 5. Discussion

Beyond regulating thermogenesis, BAT has been recently recognized as an endocrine organ that secretes various signalling factors ‘batokines’ acting locally in brown adipocytes and distant tissues/organs to regulate systemic energy metabolism [23]. Thus, BAT is viewed as a potential therapeutic target to combat obesity or related metabolic complications [81]. Growing interest has been in understanding the pleiotropic effects of a novel batokine Nrg4 [35], including its cardioprotective properties, promotion of angiogenesis, and regulation of lipid and glucose homoeostasis [24,35,82]. This systemic review provides a crucial analysis of evidence regarding the role of Nrg4 as a potential risk factor and/or biomarker for obesity, GDM, T2DM, NAFLD, and CVD.

Many studies have demonstrated that the severity of obesity and MetS were closely linked with low circulating levels [31,40,41], whereas increased Nrg4 levels were deemed as a rescue or compensatory response to metabolic dysregulations in simple obesity. Preclinical evidence showed that Nrg4 overexpression prevented high-fat diet-induced obesity, insulin resistance, and hepatic lipogenesis in mice [83,84], while Nrg4 deficiency exacerbated hepatic steatosis and insulin resistance [27]. Of note, individuals with MetS or metabolically unhealthy obesity had lower Nrg4 levels compared to those with metabolically healthy obesity without MetS [31,40,41]. Interestingly, Nrg4 levels were elevated in individuals with obesity compared to the subjects without obesity [42]. Taken together, these findings suggest that Nrg4 levels might depend on the evolution and developmental stage of metabolic disorders. It is also speculated that the increase of Nrg4 levels may reflect the compensatory response to impaired metabolic function or impaired Nrg4/ErbB4 signalling pathway due to Nrg4 receptor resistance [25]. As opposed to elevated Nrg4 levels, downregulation of Nrg4 expression in adipose tissue of mice and humans with unhealthy obesity compared to simple obesity is largely common [24,85]. This might explain the findings from most studies showing that circulating levels of Nrg4 were low or reduced in patients with diverse metabolic disorders.

Since obesity is characterized by a chronic inflammatory response, it has been demonstrated that low Nrg4 levels were negatively associated with inflammatory markers, such as hs-CRP, TNF-α, IL-6, and MCP-1 in conditions of MetS [56,67,68,70]. In adipose tissue, TNF-α may be involved in the regulation of Nrg4 level which may be one of the causes of the reduced Nrg4 expression in obesity with chronic inflammation [86]. In agreement with these findings, Nrg4 expression is repressed by pro-inflammatory signalling in adipose tissue of diet-induced metabolic disorders in mice [24]. Apart from the inflammation, decreased Nrg4 levels could be the consequence of augmented dyslipidemia, oxidative stress, and insulin resistance [68]. For example, Wang et al. [50], and Tutunchi et al. [51] found that low serum Nrg4 levels were inversely correlated with the proxies of adiposity and impaired lipid metabolism, such as BMI, WC, TG, and HOMA-IR in children and adults with NAFLD. Insulin resistance is one of the major causes of GDM and it is a hallmark of T2DM [87–89]. Indeed, several studies showed that low Nrg4 levels were negatively associated with HOMA-IR in women with GDM [56,59,61]. Zhang et al. [66] found that low serum Nrg4 levels were closely related to FPG, FPI, and HOMA-IR, suggesting Nrg4 deficiency may exacerbate the clinical manifestation of insulin resistance in newly diagnosed T2DM. In patients with T2DM and DPN, Nrg4 levels were negatively correlated with inflammation and oxidative stress markers hs-CRP, 88-iso-PGF2α, and VPT [70,71], suggesting that Nrg4 deficiency may trigger the development of atherosclerosis [67]. Other cumulative evidence showed that low Nrg4 levels were associated with increased carotid intimal thickness [33], severity of CAD [34,79], and ACS [78], advocating that Nrg4 may serve as the link between metabolic syndrome and atherosclerosis.

Although there is a growing large body of clinical evidence demonstrating the association between circulating Nrg4 levels and various metabolic diseases, there are several limitations to be acknowledged. Firstly, most of the studies were based on cross-sectional and case-control designs with a limited sample size and lack of follow-ups, while the prospective cohort studies are very scanty. Secondly, there is limited evidence on the causal relationship between circulating Nrg4 levels and the development of metabolic diseases. Thirdly, quantification of Nrg4 using ELISA which is not suitable for high-throughput screening and analysis. Thus, future studies should consider the following: (i) prospective study design covering a wider range of ethnicities, and large sample sizes are warranted to address the heterogeneity, and (ii) standardized Nrg4 quantification assays and analysis for high-throughput screening. As an alternative to the routinely used ELISAs [90,91], multiple reaction monitoring (MRM)-based mass spectrometry should be considered for future longitudinal studies because it is capable of running multiplexed assays and allows the investigation of relationships between adipokines and phenotypes or clinical parameters in large cohorts [92].

## 6. Conclusion and future perspectives

**Figure 2.**
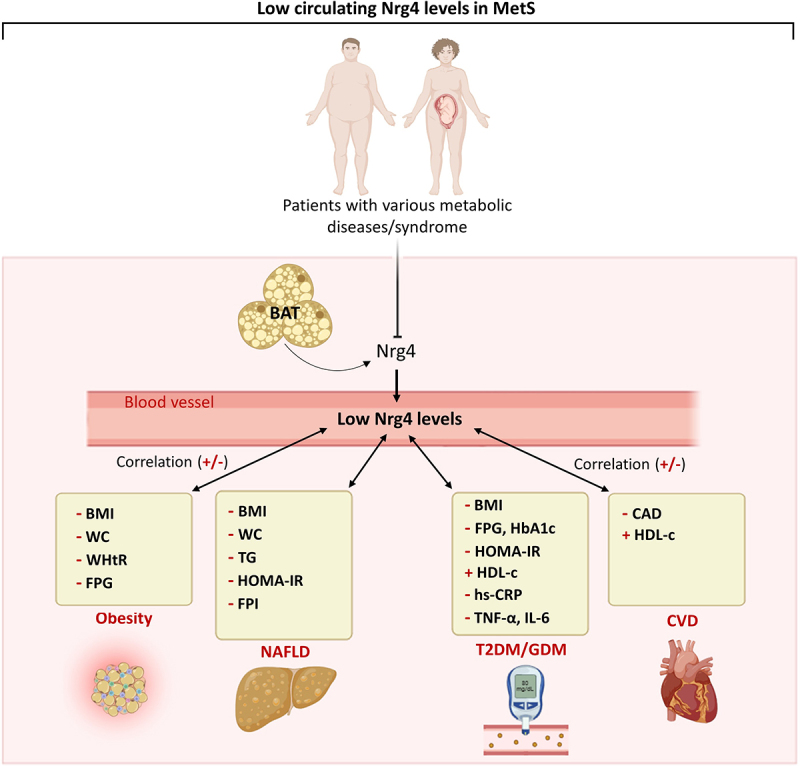
Putative model depicting the low circulating levels of neuregulin 4 (Nrg4) and its association with the markers of obesity and related metabolic diseases, including non-alcoholic diseases (NAFLD), type 2 diabetes mellitus (T2DM), and gestational diabetes mellitus (GDM), as well as cardiovascular diseases (CVD). BAT, brown adipose tissue; BMI, body mass index; CAD, coronary artery disease; FPG, fasting plasma glucose; FPI, fasting plasma insulin; HbA1c, glycated hemoglobin A1c; hdl-c, high-density lipoprotein cholesterol; hs-crp, high-sensitivity C-reactive protein; HOMA-IR, homeostatic model assessment for insulin resistance; IL-6, interleukin 6; NAFLD, non-alcoholic diseases; MCP-1, monocyte chemoattractant protein-1, Nrg4, neuregulin 4; TC, total cholesterol; TG, triglyceride; tnf-α, tumor necrosis factor alpha; WC, waist circumference; WHtR, waist-height ratio.

The current state of scientific evidence indicates that circulating Nrg4 levels are decreased in morbid obesity, and it is associated with the indices of metabolic diseases, such as obesity, GDM, T2DM, NAFLD, and CVD (Figure 2). Emerging observational studies also suggest that low circulating levels of Nrg4 can be a potential risk factor that either directly or indirectly contributes to the incidence of obesity-related metabolic diseases, thus, it is proposed that Nrg4 could be a useful biomarker for predicting severe consequences of obesity. However, well-designed longitudinal studies covering a wider range of ethnicities and large sample sizes are required to elucidate the heterogeneity of available studies. Such prospective cohort-designed studies are necessary to accumulate more evidence about the causality relationship between low circulating Nrg4 levels and obesity-related metabolic defects. Further research should also provide knowledge regarding the development of new therapeutics targeting Nrg4.

Alt Text. The diagram shows two individuals with metabolic syndrome including a pregnant woman, there is a blunt arrow showing inhibition of Nrg4 release from its primary source brown adipose tissue (BAT). At the bottom, double-sided arrows show the correlation between low plasma Nrg4 levels (in the blood vessel) and metabolic diseases including obesity, non-alcoholic diseases (NAFLD), type 2 diabetes (T2DM), and gestational diabetes mellitus (GDM), as well as the cardiovascular disease (CVD).

## Supplementary Material

Supplementary material 2_Quality Assessment.docx

Supplementary material 1_PRISMA checklist.doc

## Data Availability

Data sharing is not applicable to this article as no new data were created or analysed in this study.

## Abbreviations

AST: aspartate aminotransferase
ALT: alanine transaminase
BAT: brown adipose tissue
BMI: body mass index
CAD: coronary artery disease
CVD: cardiovascular diseases
BP: blood pressure
Egfr: estimated glomerular filtration rate
FPG: fasting blood glucose
FPI: fasting plasma insulin
GDM: Gestational diabetes mellitus
GGT: gamma-glutamyl transpeptidase
HbA1c: glycated haemoglobin A1c
HDL-c: high-density lipoprotein cholesterol
HC: hip circumference
hs-CRP: high-sensitivity C-reactive protein
HTN: hypertension
HOMA-IR: homoeostatic model assessment for insulin resistance
IL-6: interleukin 6
LDL-c: low-density lipoprotein
NAFLD: non-alcoholic diseases
Nrg4: neuregulin 4
NOS: Newcastle-Ottawa Scale
SBP: systolic blood pressure
T2DM: type 2 diabetes mellitus
TC: total cholesterol
TG: triglyceride
TNF-α: tumour necrosis factor
TyG: triglyceride-glucose index
VPT: vibration perception threshold
WC: waist circumference
