# Supplementary material for: Low circulating levels of neuregulin 4 as a potential biomarker associated with the severity and prognosis of obesity-related metabolic diseases: a systematic review
Source: Adipocyte. 2024 Aug 20;13(1):2390833. doi: 10.1080/21623945.2024.2390833 (PMC11340757; doi:10.1080/21623945.2024.2390833)
Supplement: Supplementary material 2_Quality Assessment.docx [file KADI_A_2390833_SM9760.docx]

**Supplementary Table 1.** Outcomes of Newcastle-Ottawa Scale score in cohort study

| Year; Author | Selection | | | | Comparability | Outcome | | | Total score  (0-9) |
| --- | --- | --- | --- | --- | --- | --- | --- | --- | --- |
|  | Representativeness of the cohort | Selection of the non-exposed cohort | Ascertainment of exposure | Outcomes were not present at the beginning of the study |  | Assessment of the outcome | Follow-up duration | Adequacy of follow-up |  |
| Kralisch et al., 2018 | ★ | ★ | - | ★ | ★ | ★ | ★ | ★ | 8 |
| Ding et al. 2023 | ★ | ★ | - | ★ | ★ | ★ | ★ | ★ | 8 |

Article quality was assessed as follows: low quality = 1–3; moderate quality = 4–6; high quality = 6–9.

**Supplementary Table 2.** Outcomes of Newcastle-Ottawa Scale score in case-control

| Year; Author | Selection | | | | Comparability | Outcome | | | Total score  (0-9) |
| --- | --- | --- | --- | --- | --- | --- | --- | --- | --- |
|  | Case definition | Representativeness of the cases | Selection of controls | Definition of controls |  | Ascertainment of exposure | Method of ascertainment | Non-response rate |  |
| Dai et al. 2015 | ★ | ★ | ★ | ★ | ★★ | ★ | - | - | 7 |
| Kang et al. 2016 | ★ | ★ | ★ | - | ★★ | ★ | ★ | - | 8 |
| Chen et al. 2017 | ★ | ★ | ★ | ★ | ★★ | ★ | ★ | - | 8 |
| Kralisch et al., 2018 | ★ | ★ | ★ | ★ | ★★ | ★ | ★ | - | 8 |
| Rahimzadeh et al. 2020 | ★ | ★ | ★ | ★ | ★★ | ★ | ★ | - | 8 |
| Tutunchi et al. 2021 | ★ | ★ | ★ | ★ | ★★ | ★ | ★ | - | 8 |
| Martínez et al. 2022 | ★ | ★ | ★ | ★ | ★★ | ★ | - | - | 7 |
| Cindoglu et al. 2023 | ★ | ★ | ★ | ★ | ★★ | ★ | - | - | 7 |
| Al-Bayati and Saleh 2023 | ★ | ★ | ★ | ★ | ★★ | ★ | ★ | - | 7 |
| Zhong et al. 2023 | ★ | ★ | ★ | ★ | ★ | ★ | ★ | - | 7 |

Article quality was assessed as follows: low quality = 1–3; moderate quality = 4–6; high quality = 6–9.

**Supplementary Table 3.** Outcomes of Newcastle-Ottawa Scale score in cross-sectional studies

| Year; Author | Selection | | | | Comparability | Outcome | | Total score  (0-9) |
| --- | --- | --- | --- | --- | --- | --- | --- | --- |
|  | Representativeness of the sample | Sample size | Non-respondents | Ascertainment of exposure |  | Assessment of the outcome | Statistical test |  |
| Cai et al., 2016 | ★ | ★ | - | ★★ | ★ | ★★ | ★ | 8 |
| Jiang et al. 2016 | ★ | ★ | - | ★★ | ★ | ★★ | ★ | 8 |
| Zhang et al., 2017 | ★ | ★ | - | ★ | ★ | ★ | ★ | 6 |
| Kurek et al., 2017 | ★ | ★ | - | ★ | ★ | ★★ | ★ | 7 |
| Yan et al., 2017 | ★ | ★ | - | ★ | ★ | ★★ | ★ | 7 |
| Yan et al., 2018 | ★ | ★ | - | ★ | ★ | ★★ | ★ | 7 |
| Yan et al. 2019 | ★ | ★ | - | ★★ | ★ | ★★ | ★ | 8 |
| Wang et al., 2019 | ★ | ★ | - | ★ | ★ | ★★ | ★ | 7 |
| Yan et al., 2020 | ★ | ★ | - | ★★ | ★ | ★★ | ★ | 8 |
| Tian et al. 2019 | ★ | ★ | - | ★ | ★ | ★★ | ★ | 7 |
| Kocak et al., 2019 | ★ | ★ | ★ | ★★ | ★ | ★ | ★ | 8 |
| Su-su et al. 2019 | ★ | ★ | - | ★ | ★ | ★ | ★ | 6 |
| Kocak et al., 2020 | ★ | ★ | - | ★ | ★ | ★ | ★ | 6 |
| De Munck et al., 2021 | ★ | ★ | - | ★★ | ★ | ★★ | ★ | 8 |
| Gou et al., 2021 | ★ | ★ | - | ★★ | ★ | ★★ | ★ | 8 |
| Zhang et al., 2021 | ★ | ★ | - | ★★ | ★ | ★★ | ★ | 8 |
| Attique et al., 2022 | ★ | ★ | - | ★★ | ★ | ★★ | ★ | 8 |
| Li et al., 2022 | ★ | ★ | - | ★★ | ★ | ★ | ★ | 7 |
| Alipoor et al., 2023 | ★ | ★ | - | ★★ | ★ | ★ | ★ | 7 |

Article quality was assessed as follows: low quality = 1–3; moderate quality = 4–6; high quality = 6–9
